# Intersectionality: Mapping Critical Relations for Quality in Long-Term Care Research

**DOI:** 10.1093/geroni/igaa057.127

**Published:** 2020-12-16

**Authors:** Katie Aubrecht, Ivy Bourgeault, Tamara Daly

**Affiliations:** 1 St. Francis Xavier University, Antigonish, Nova Scotia, Canada; 2 University of Ottawa, Ottawa, Ontario, Canada; 3 York University, Toronto, Ontario, Canada

## Abstract

Intersectionality is a useful method (Lutz, 2015) for interdisciplinary long-term care (LTC) research to advance a more critical understanding of how experiences of quality are shaped by mutually reproducing social divisions, identities and relations of power that shape LTC. This paper discusses insights from the “Mapping Care Relationships” stream of the Seniors – Adding Life to Years (SALTY) project, a pan-Canadian program of research examining clinical, social and policy perspectives on quality in LTC. “Mapping Care Relationships” mapped how promising approaches to care relationships are organized and experienced in LTC. From January 2018-August 2019 our team of nine researchers conducted rapid ethnographies in eight nursing homes, two in each of four provinces across Canada. We purposively observed and interviewed workers from a wide variety of positions and backgrounds, informed by an intersectionality approach. We traced how promising approaches in person-centred dementia care (PCDC) in particular may reify the subordinated status of care workers (some more than others) and reinforce inequities within LTC systems. In multiple LTC homes, front-line care workers described experiencing physical and emotional harm in care relationships with residents which caused them distress. However, consistent with a PCDC approach, the harm was attributed to ‘behaviours’ clinically symptomatic of dementia. In framing power differentials from a medical perspective, PCDC makes it possible to interpret harmful experiences as 'part of the job’ and something workers should know to expect, prevent, avoid, redirect, or ignore. Lutz, H. (2015). Intersectionality as method. DiGeSt. Journal of diversity and gender studies, 2(1-2), 39-44.

